# Fibrocyte measurement in peripheral blood correlates with number of cultured mature fibrocytes in vitro and is a potential biomarker for interstitial lung disease in Rheumatoid Arthritis

**DOI:** 10.1186/s12931-017-0623-9

**Published:** 2017-07-18

**Authors:** Søren Andreas Just, Hanne Lindegaard, Eva Kildall Hejbøl, Jesper Rømhild Davidsen, Niels Bjerring, Søren Werner Karlskov Hansen, Henrik Daa Schrøder, Inger Marie Jensen Hansen, Torben Barington, Christian Nielsen

**Affiliations:** 1 Department Rheumatology, Odense University Hospital, Odense, Denmark; 2 Department Pathology, Odense University Hospital, Odense, Denmark; 3 South Danish Center for Interstitial Lung Diseases, Odense University Hospital, Odense, Denmark; 4 Department Respiratory Medicine, Odense University Hospital, Odense, Denmark; 5 Institute of Molecular Medicine, University of Southern Denmark, Odense, Denmark; 6 Department Medicine, Svendborg Hospital, Odense University Hospital, Svendborg, Denmark; 7 Department Clinical Immunology, Odense University Hospital, Odense, Denmark; 8 Odense Patient data Explorative Network (OPEN), Odense University Hospital, Odense, Denmark

**Keywords:** Rheumatoid arthritis, Interstitial lung disease, Fibrocytes

## Abstract

**Background:**

Interstitial lung disease (ILD) can be a severe extra-articular disease manifestation in Rheumatoid Arthritis (RA). A potential role of fibrocytes in RA associated ILD (RA-ILD) has not previously been described. We present a modified faster method for measuring circulating fibrocytes, without intracellular staining. The results are compared to the traditional culture method, where the number of monocytes that differentiate into mature fibrocytes in vitro are counted. The results are following compared to disease activity in patients with severe asthma, ILD, RA (without diagnosed ILD) and RA with verified ILD (RA-ILD).

**Method:**

CD45^+^ CD34^+^ CD11b^+^ (7-AAD^−^ CD3^−^ CD19^−^ CD294^−^) cells were isolated by cell sorting and stained for pro-collagen type 1. Thirty-nine patients (10 RA, 9 ILD and 10 with severe asthma, 10 with RA-ILD) and 10 healthy controls (HC) were included. Current medication, disease activity, pulmonary function test and radiographic data were collected. Circulating fibrocytes were quantified by flow cytometry. Peripheral blood mononuclear cells were isolated and cultured for 5 days and the numbers of mature fibrocytes were counted.

**Results:**

90.2% (mean, SD = 1.5%) of the sorted cells were pro-collagen type 1 positive and thereby fulfilled the criteria for being circulating fibrocytes. The ILD and RA-ILD groups had increased levels of circulating fibrocytes compared to HC (*p* < 0.05). Levels of circulating fibrocytes correlated overall to number of monocytes that subsequently in vitro differentiated to mature fibrocytes (*r* = 0.81, *p* < 0.001). RA patients with pathologically reduced diffusion capacity for carbon monoxide adjusted for hemoglobin (DLCO_c_) in both the RA and in the combined RA + RA-ILD group, had significantly higher levels of both circulating and number of cultured mature fibrocytes (both *p* < 0.05). In both groups, the level of circulating fibrocytes and number of mature fibrocytes in culture also correlated to a reduction in DLCO_c_ (*r* = −0.61 an *r* = −0.58 both *p* < 0.05).

**Conclusions:**

We presented a fast and valid method for measuring circulating fibrocytes using flow cytometry on lysed peripheral blood. Further, we showed for the first time, that the level of circulating fibrocytes correlated with the number of peripheral blood mononuclear cells, that differentiated into mature fibrocytes in vitro. Reduced DLCO_c_ was correlated with high levels of circulating and mature fibrocytes in RA, which have not been reported previously. In such, this study suggests that fibrocytes may exhibit an important role in the pathogenesis of RA-ILD, which requires further clarification in future studies.

**Trial registration:**

ClinicalTrials.gov:NCT02711657, registered 13/3–2016, retrospectively registered.

**Electronic supplementary material:**

The online version of this article (doi:10.1186/s12931-017-0623-9) contains supplementary material, which is available to authorized users.

## Background

Interstitial lung diseases (ILDs) are a heterogeneous group of Diffuse Parenchymal Lung Diseases (DPLD) that may be idiopathic or secondary to a known etiology. In Rheumatoid Arthritis (RA) 10–20% of all patients present with pulmonary manifestations as part of RA associated ILD (RA-ILD). RA-ILD has limited treatment options and is attributable to nearly 10% of deaths in RA patients [1].

The precursor cell type for the lung myofibroblasts, which in ILD are persistently activated and produce excessive amounts of extracellular matrix fibrosis components, has yet to be identified [2, 3]. Bone marrow-derived cells, called fibrocytes, are believed to originate from a monocyte-derived precursor, and fibrocyte levels are increased both in peripheral blood and in the affected tissue in fibrotic diseases [4]. Fibrocytes are defined as cells expressing both hematopoietic (e.g. CD45, CD34), myeloid (e.g. CD11b) and stromal markers (e.g. pro-collagen type 1) [5]. Fibrocytes migrate to sites of inflammation or tissue damage and transform into spindle shaped fibroblast-like cells classified as the mature fibrocyte [2]. The transformation is controlled by T-cell interaction and a wide range of inducing or inhibiting chemokines and cytokines [6, 7]. The mature fibrocytes secrete extracellular matrix fibrosis components like collagen I/III and fibronectin, and the cells are naturally involved in both tissue repair and fibrosis [4]. Notably, collagen is a central component in fibrosis matrix during pathophysiological conditions. Further, elevated fibrocyte levels both circulating and in lung biopsies have been reported in patients with idiopathic pulmonary fibrosis (IPF) [8–10]. In murine models, development of lung fibrosis is directly associated with the influx of fibrocytes to the lung [2, 11]. Moreover, it has been demonstrated that adaptive infusion of fibrocytes worsened disease activity in murine disease models of lung and kidney fibrosis and also in collagen induced arthritis [12]. Additionally, in patients with severe asthma, disease activity has been found to correlate with the levels of circulating and intrapulmonary fibrocytes [13–15]. In summary, current knowledge indicates that circulating fibrocytes could be one of the precursor cells of the lung fibroblasts and myofibroblasts with a central role in fibrotic ILD pathogenesis. However, whether fibrocytes are involved in RA-ILD is currently unknown.

The used method for culturing mature fibrocytes from isolated peripheral blood mononuclear cells (PBMC), using serum free medium (SFM) has previously been validated and used in several studies [7, 16–18]. Culturing is often done with an agent stimulating fibrocyte differentiation, in particular interleukin 4 (IL-4). IL-4 is produced by T helper cell type 2 (Th2) cells. Elevated Th2 cell levels are observed in early RA (blood and synovial fluid), IPF (blood and lung), and asthma (blood and lung) [19–21].

Circulating fibrocytes have traditionally been identified among PBMCs by measurement of expression levels of extracellular hematopoietic cell surface markers (cluster of differentiation (CD); typically, CD45 and/or CD34) combined with cell permeabilization and labeling for intracellular mesenchymal markers (e.g. collagen type 1/pro-collagen type 1/vimentin) [22]. However, this methodology is time consuming and expensive. Also, it contains several washing steps to isolate PBMCs, uses cell permeabilization, fails to provide absolute fibrocyte concentration and results in non-viable cells making it impossible to analyze their differentiative capacity [13]. Recently, a method for measuring fibrocytes in peripheral blood by flow cytometry using only cell surface markers followed by red blood cell lysis has been introduced and validated [13]. This method enumerates CD45^+^ CD34^+^ CD11b^+^ (CD3^−^ CD19^−^ CD20^−^ CD294^−^ CD115^−^ CD16^−^ 7AAD^−^) cells, and the validation experiments by Bianchetti et al. found the cells to contain intracellular collagen when stimulated with endothelin 1 (ET-1), and thereby fulfilling the definition for being fibrocytes [13].

Mature tissue resident spindle shaped fibrocytes and circulating fibrocytes are two distinct entities. Thus, it could be informative to measure both concomitantly. We hypothesized that levels of circulating fibrocytes can be measured, using surface markers on lysed blood cells only, and that these levels correlate with levels of mature cultured fibrocytes.

The aim of this study was to establish a fast and reliable method for quantification of circulating fibrocytes and evaluate whether the fibrocyte levels correlated to disease severity in patients with fibrotic ILDs (IPF and non-specific interstitial pneumonitis (NSIP)), severe asthma, RA (not diagnosed with ILD) and RA with verified ILD (RA-ILD).

## Methods

### Study design, setting and study cohort

We performed a single center case-based cross-sectional study at Odense University Hospital (OUH) with inclusion of 10 blood donors from the Department of Clinical Immunology, 10 patients receiving omalizumab (Xolair, Novartis) for severe asthma, 9 patients with fibrotic ILD (IPF or NSIP) from the Department of Respiratory Medicine, 10 patients with RA and 10 RA patients with verified RA-ILD from the Department of Rheumatology. All study subjects were included as part of their regular follow-up. RA patients fulfilled the EULAR/ACR 2010 criteria for RA and Disease Activity Score in 28 joints combined with CRP value (DAS28CRP) were recorded. Further Rheumatoid factor (RF) and cyclic citrullinated peptide antibody (anti-CCP) value was recorded. Data on peak flow, Asthma Control Test (ACT) score, and pulmonary function tests (PFT) were recorded on severe asthma, ILD and RA-ILD patients. Diagnoses regarding severe asthma, ILD and RA-ILD were confirmed on prior multi-disciplinary discussion conferences with presence of pulmonologists, rheumatologists, radiologists and pathologists. No criteria regarding disease duration or disease activity were set for enrollment.

For the establishment of the method, blood from healthy blood donors was used, with only information regarding age and sex registered.

### Flow cytometry

**Fig. 1:**
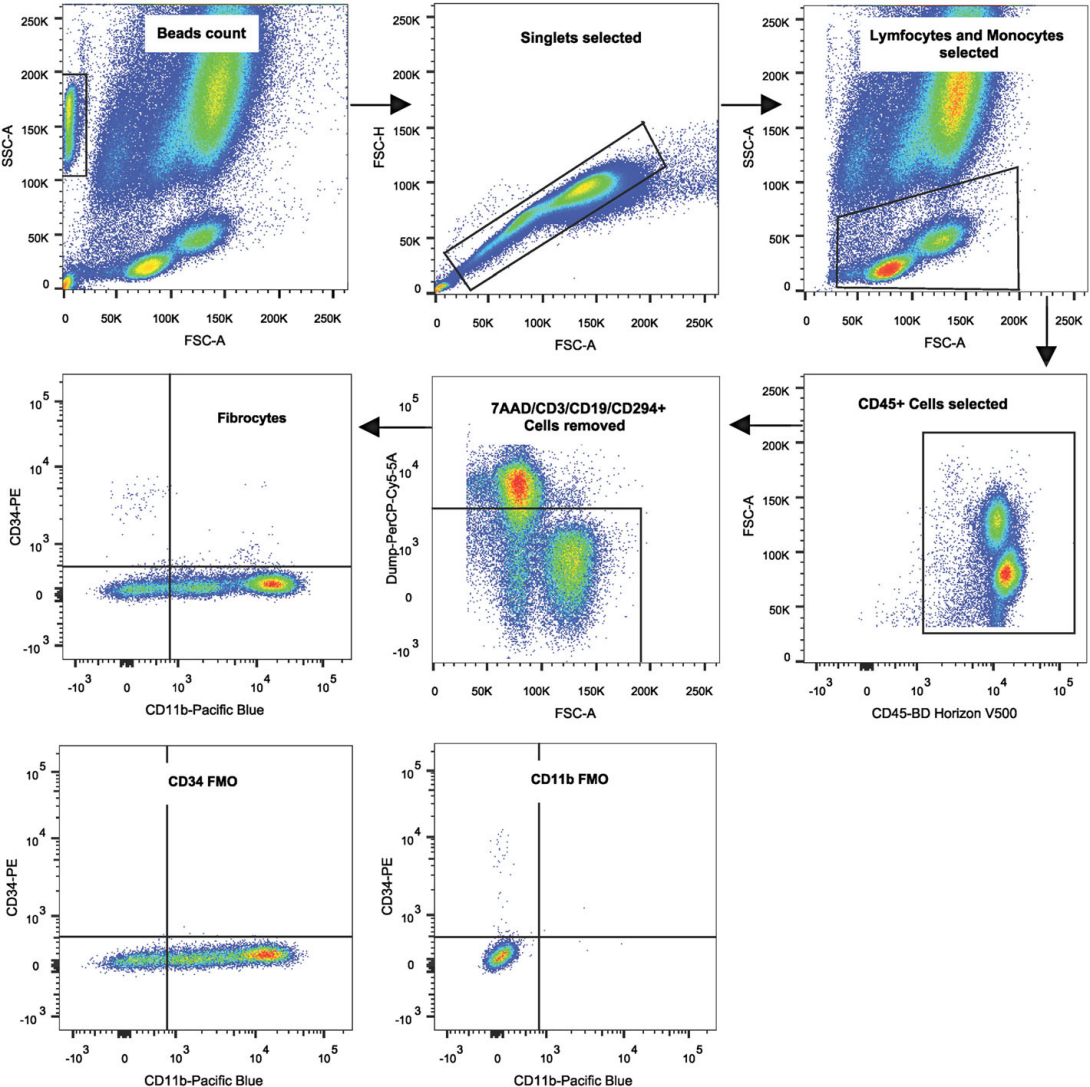
Gating strategy for quantification of fibrocytes using lysed peripheral blood. Representative flow cytometry analysis showing the gating strategy. Population was initially gated on the basis of the forward side scatter characteristics and debris were eliminated, then lymphocyte and monocytes were selected, the CD45^+^ cells were gated in the plot “CD45-V500” versus forward scatter. Hereafter unwanted cells as B-Cells (CD19^+^), T-Cells (CD3^+^), basophils and eosinophils (CD294^+^) and dead cells (7AAD^+^) were removed using a PerCP-Cy5-5A dump channel. Finally, the CD45^+^ CD34^+^ Cd11b^+^ fibrocyte population was measured in the “CD11b-Pacific blue versus CD34-PE” plot. Fluoroscence minus one (FMO) controls are shown at the bottom

Twenty milliliter peripheral blood was collected in ethylenediaminetetraacetic acid (EDTA). Hereafter, a slightly modified method of Bianchetti et al. was used [13]. Briefly, 100 μL of whole blood were transferred to a Trucount absolute counting tube (BD Bioscience, San Jose, USA) and labelled with the following monoclonal antibodies: CD45-V500C (dose 5 μL Clone 2D1), CD34-PE (dose 5 μL Clone 581), CD11b-Pacific Blue (dose 5 μL Clone ICRF44), Via-Probe (7-AAD) (dose 2 μL), CD3-PE-Cy5 (dose 2.5 μL Clone HIT3a), CD19-PerCP-Cy5.5(dose 2.5 μL Clone HIB19), CD294-PerCP-Cy5.5 (dose 2.5 μL Clone BM16) (All BD Bioscience, San Jose, USA), all titrated for no-wash staining. After 20 min of dark incubation at room temperature, 2 ml of High Yield Lyse Solution (Thermo Fisher Scientific, Waltham, USA) were added for red cell lysis and tubes were stored for 10 min in the dark. Immediately hereafter, the sample was analyzed using a BD FACSCanto II (4 laser) flow cytometer, which was adjusted using BD Cytometer Setup and Tracking Beads. Compensation settings were performed using BD CompBead antibody-capturing particles (BD Bioscience, San Jose, USA) and the automatic compensation setup tool in the BD FACS Diva software. Fluorescence-minus-one controls were used for accurate definition of cells that had fluorescence above background levels. Relevant isotype controls for CD45-V500C (dose 5 μL Clone X40), CD34-PE (dose 5 μL Clone MOPC-21) and CD11b-Pacific Blue (dose 5 μL Clone MOPC-21) (all BD Bioscience, San Jose, USA) were used in the initial setup and frequently between tests. Data was acquired using BD FACSDiva software (BD Bioscience, San Jose, USA) and analyzed on FlowJo version 10 (Treestar, Ashland, USA). The gating strategy is described in Fig. 1.

For validation, coefficient of variation (CV%) was measured using blood from a healthy individual by measuring six independently prepared samples from the same blood tube. Further, CV% was assessed by measuring on the same stained sample 5 times. To test the effect of storing the blood sample for 24 h at room temperature on the fibrocyte count measurement, 4 samples were prepared from a stored blood tube and stained. Further, the effect of storing for 24 h was assessed for the stained and lysed samples. In the following the measured population of CD45^+^ CD34^+^ CD11b^+^ (7-AAD^−^ CD3^−^ CD19^−^ CD294^−^) cells will be referred to as CD45^+^ CD34^+^ CD11b^+^ cells.

CD45^+^ CD34^+^ CD11b^+^ cells were further tested for the expression of the following surface markers: CXCR4-APC (dose 5 μL Clone 12G5), CD115-Alexa Fluor 647 (dose 5 μL Clone 94D21E4), CD16-FITC (dose 7.5 μL Clone B73.1) and CD20-PerCP-Cy5.5 (dose 1.25 μL Clone 2H7) (all BD Bioscience, San Jose, USA).

### Culturing mature fibrocytes

PBMCs were cultured as previously described [16]. Briefly, PBMCs were isolated from 20 ml of peripheral EDTA blood using Lymphoprep (StemCell, Grenoble, France) according to manufacturer’s instructions. The culture media consisted of the following products for the production of a total of 50 ml: 47 ml of FibroLife (LifeLine Cell Technology, Frederick, USA), 0.5 ml of HEPES (10 mM), 0.5 ml Non-essential amino acids (NEAA), 0.5 ml of Sodium pyruvate (1 mM), 0.5 ml of Glutamine (2 mM), 0.5 ml of Pen-Strep (Penicillin (100 U/ml) and Streptomycin (100 μg/ml)), and 0.5 ml of ITFS-3 (All Sigma-Aldrich, St. Louis, USA) and 5 ng/ml IL-4 (CellSystems, Troisdorf, Germany). A monocyte, lymphocyte, granulocyte and eosinophil count was performed using PBMCs (Sysmex, Kobe, Japan). Hereafter cells were adjusted to 1 × 10^6^ cells/ml culture media. Flat bottom 24-well plates (Nunc, Thermo Fisher Scientific) were used in combination with glass coverslips (Thermo Fisher Scientific) placed at the bottom of each well. Ten wells were each filled with 200 μl of cell suspension and supplemented with a further 500 μl of culture media. The plate was placed in a humidified incubator (37 °C, 5% CO_2_) for 5 days. Non-adherent cells (e.g. T and B-cells) were discarded and the remaining cells were harvested, by removing the coverslips, washing twice in PBS and drying it. Before staining the coverslips were mounted on glass slides.

### Quantification of fibrocytes

**Fig. 2:**
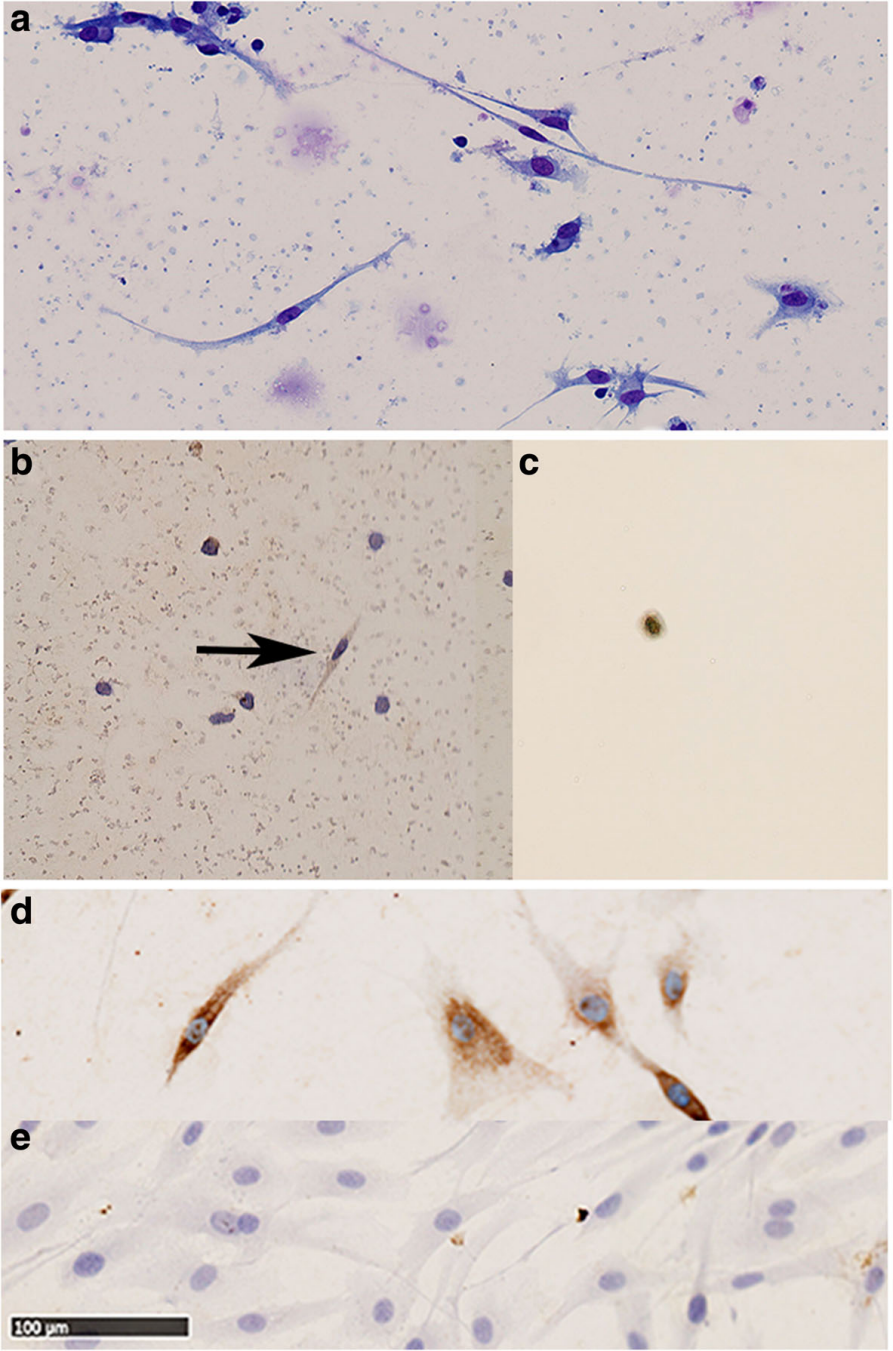
Mature and circulating fibrocytes. Appearance of fibrocytes in **a** PBMCs cultured for 5 days in serum free medium; giemsa stain. **b** Cultured PBMCs. The arrow points at a pro-collagen type 1 positive cell. **c** Pro-collagen type 1 positive CD45^+^CD34^+^CD11b^+^ sorted cell from an RA patient. **d** Cultured fibroblast used as positive control for the pro-collagen type 1 stain. **e** IgG isotype control. The scale bar represents 100 μm

Two coverslips were Giemsa stained, scanned using NanoZoomer 2.0-HT (Hamamatsu, Hamamatsu, Japan) and cells were counted using NDPIviewer (Hamamatsu). Mature fibrocytes were defined as spindle shaped cells with a minimum length of 40 μm (See Fig. 2a). For positive cell counting, eight fields on each of two separate coverslips were randomly selected and counted for fibrocytes. The range of counted cells per person was from 7 to 148 fibrocytes. From this number, an estimate of the total number of fibrocytes in the well was calculated and the fibrocyte counts from two different wells from the same patient were averaged to give the fraction of PBMCs that differentiated to fibrocytes. By using the differential count performed on the isolated PBMCs before culture, the number of fibrocytes per 10^5^ monocytes added to the well was calculated. For validation of the fibrocyte culture method, the number of cells counted on the two different wells from the same patient was correlated. The stained samples from the RA-ILD patients were counted manually with a microscope, using the same method as described above.

### Immunocytochemistry

Immunocytochemistry was performed on cultured fibrocytes on coverslips. Staining was performed using the PowerVision+/DAB detection system. The primary antibody used was Anti-Procollagen Type 1 antibody (clone 2Q576, Abcam, Cambridge, UK) at a dilution of 1:150.

### Fluorescence activated cell sorting (FACS)

Cell sorting was done to determine whether the used antibody panel identified cells that express pro-collagen type 1. Twenty milliliter of peripheral blood were drawn from two RA and two asthma patients and PBMCs were isolated. Firstly, CD34^+^ cells were isolated using positive selection magnetic beads (StemCell, Grenoble, France). The process was done according to manufacturer’s instructions, with one modification. The RosettaSep cocktail was not added to avoid removing CD16^+^ cells, as a fraction of fibrocytes has been reported be CD16^+^ (discussed later) [23].

The isolated CD34^+^ cells were diluted in Hanks balanced salt solution, with human albumin and EDTA (0.5 mg of HSA/ml, 2 mM EDTA, Sigma-Aldrich, St. Louis, USA). The cells were stained for CD34 (dose 20 μL clone 8G12) and CD11b, CD45 CD3, CD19, CRTH2 and with 7 AAD (same dose, clones and firm as described under Flow cytometry). Cells were aseptically sorted on a BD FACSAria cytometer (BD Bioscience, San Jose, USA) using the BD FACSDiva software and the gating strategy as described in Fig. 1. The CD45^+^ CD34^+^ cells were divided into CD11b^+^ and CD11b^−^ cells and placed in flat bottomed wells with a glass coverslip placed at the bottom. Fibrocytes were matured wit IL-4 as above, but only cultured for 2 days. Purity of sorted cells was evaluated by flow cytometry and the CD34 isolation negative selection rest product. Further information on flow cytometry, cell culture and cell sorting procedure, is presented in Additional file 1: Table S1.

### Disease activity

Disease activity, current medication and PFTs results were collected from the patients’ medical records. C-reactive protein (CRP), hemoglobin, leukocyte differential count, creatinine and liver function tests were measured at time of inclusion (+/− 1 day) as part of routine monitoring of disease activity. Elevated levels were defined as leucocytes >10 × 10^9^/L, neutrophils >7 × 10^9^/L, lymphocytes > 4 × 10^9^/L, monocytes >0.7 × 10^9^/L, eosinophils > 0.5 × 10^9^/L, basophils >0.2 × 10^9^/L and for CRP >10 mg/L.

Thoracic imaging data (i.e. chest X-ray and/or high-resolution computed tomography (HRCT)) performed within 2 years from the date of obtaining blood test was registered. A pathological hemoglobin corrected diffusion capacity for carbon monoxide (DLCO_c_) was defined as below 80% of predicted.

### Statistics

Categorical variables were presented as numbers and percentages, and continuous variables as medians with 25th -75th percentile values (P25-P75). When two groups were compared Fisher’s exact test for categorical data and Mann-Whitney U test for continuous data was used. Correlations were examined with Spearman’s correlation. Comparisons between multiple groups were done with Kruskal-Wallis test followed by Dunn’s post hoc test. Statistical significance was identified as *p*-value <0.05. Data were analyzed on Stata version 14.0 (StataCorp, Texas, USA).

## Results

### Circulating Fibrocytes

The purity of the sorted CD45^+^ CD34^+^ CD11b^+^ cells was 86%. At average 90.2% (SD ±1.5%) of the CD45^+^ CD34^+^ CD11b^+^ cells were pro-collagen type 1 positive compared to only 6.3% (SD ±4.6%) of the corresponding CD45^+^ CD34^+^ CD11b^−^ cells were procollagen type 1 positive (Fig. 2). The CD45^+^ CD34^+^ CD11b^+^ cell population thereby fulfilled the criteria of circulating fibrocytes.

The frequencies of CD20, CD115, CXCR4 and CD16 expression on the CD45^+^ CD34^+^ CD11b^+^ cell population were 1, 3, 75, and 45% respectively (Additional file 1: Table S2).

Repeated measurement of the sample taken the same day from a healthy donor gave a coefficient of variation (CV%) on 9.1%, with a median of 1.4 fibrocytes/μL (P25-P75 1.2–1.5). Repeated measurement on the same sample after 24 h at room temperature gave a CV% of 9.4% with a median of 1.4 fibrocytes/μL (P25-P75 1.2–1.6). No statistically significant difference between median fibrocyte values on the day of sample collection and after 24-h storage was found (*P* = 0.8). The lysed and stained samples from the day of sample collection were stored for 24 h in a refrigerator and re-measured resulting in a CV% of 14% with a median of 1.2 fibrocytes/μL (P25-P75 1.1–1.4), without a significant day to day difference (*P* = 0.3).

### Cultured fibrocytes

**Fig. 3:**
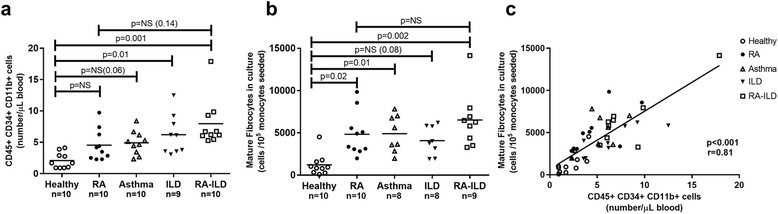
Levels of CD45^+^ CD34^+^ CD11b^+^ cells and number of mature fibrocytes after 5 days in culture. **a** Comparison of CD45^+^ CD34^+^ CD11b^+^ cell levels. NS = not significant. **b** Comparison of number of mature fibrocytes after 5 days in culture. **c** Correlation between CD45^+^ CD34^+^ CD11b^+^ cells in blood measured by flow cytometry and number of mature fibrocytes after 5 days in culture

The mean CV% among all patients and healthy controls of the number of mature fibrocytes between the two coverslips was 14.4%. This was based on 35 double enumerations, the RA-ILD group was not included, and further missing 4 double enumerations due to 3 patients (2 severe asthma and 1 ILD), for which culture failed and two asthma patients where only one coverslip could be counted. Median numbers of counted mature fibrocytes are presented in Table 1. All groups except the ILD group (*p* = 0.08) had significantly increased levels of mature fibrocytes compared to HC, while there were no significant differences in levels between the different patient groups (See Fig. 3b).

**Table 1:** Level of circulating and mature fibrocytes according to disease group

	Control	RA	Severe asthma	IPF/NSIP	RA-ILD
	*n* = 10	*n* = 10	*n* = 10	*n* = 5/*n* = 4	*n* = 10
Flow cytometry					
2003CD45^+^CD34^+^CD11b^+^ cells/μL median (P25-P75)	1.69 (0.94–2.89)	3.84 (2.52–6.25)	4.88 (3.44–6.16)	6.13 (3.58–7.93)	6.45 (5.89–9.44)
Cultured mononuclear cells					
Fibrocytes/10^5^ monocytes median (P25-P75)	887 (331–1590)	4140 (3069–5557)	4634 (3213–6881)	3897 (2570–5809)	6266 (3946–7437)

Cells presented in counted numbers and as medians with 25th- and 75th percentiles in brackets. Culture of mononuclear cells failed from 4 patient samples (2 Severe Asthma, 1 ILD, 1 RA-ILD)

### Correlation between circulating and cultured fibrocytes

Baseline demographic and clinical characteristics of study participants are presented in Table 2.

**Table 2:** Demographic and clinical characteristics of study participants

	Control	RA	Asthma	IPF/NSIP	RA-ILD
	*n* = 10	*n* = 10	*n* = 10	*n* = 5/*n* = 4	*n* = 10
Age, years, median (P25-P75)	56 (51–59)	58 (49–66)	50 (36–56)	75 (66–78)	70 (55–75)
Female sex, n (%)	4 (40)	7 (70)	3 (30)	4 (44)	6 (60)
Current/former/never smokers (%)^1^	NA	20/10/70	10/20/70	0/78/22	20/70/10
Blood sample results					
Hemoglobin, mmol/L, median (P25-P75)	9.1 (8.8–9.5)	7.8 (7.2–8.8)	9.1 (8.7–9.7)	8.5 (7.8–8.9)	8.25 (8–8.5)
CRP, mg/L, median (P25-P75)	NA	5.4 (2.7–16)	3.2 (1.8–4.7)	2.3 (2–4.4)	8 (1.9–11)
Leucocytes, 10^9^/L, median (P25-P75)	5.3 (4.6–5.8)	6.8 (5.9–9.6)	8.5 (8.1–9.1)	9.6 (8.2–11)	11.5 (6.7–14.5)
Monocytes, 10^9^/L, median (P25-P75)	0.5 (0.4–0.6)	0.6 (0.5–0.7)	0.6 (0.5–0.8)	0.8 (0.6–1.1)	0.8 (0.7–1.0)
Radiological signs compatible with interstitial lung disease and/or lung fibrosis^2^					
Chest X-ray (n/N)	0/0	1/7	0/6	9/9	6/10
High Resolution CT (n/N)	0/0	0/0	0/7	9/9	10/10
Pulmonary Function Test					
Lungfunction performed (n/N)	0/0	10/10	10/10 ^3^	9/9	10/10 ^4^
FVC (% predicted), median (P25-P75)	NA	108 (103–16)	84 (70–99)	78 (66–90)	NA
TLC (% predicted), median (P25-P75)	NA	99 (94–103)	NA	83 (64–93)	NA
FEV_1_ (% predicted), median (P25-P75)	NA	103 (92–112)	71 (61–81)	78 (72–85)	70 (39–86)
DLCO_c_ (% predicted),median (P25-P75)	NA	79 (67–89) ^5^	NA	36 (31–37)	58 (46–73)
Asthma patients					
GINA score (Average) ^6^			5		
ACT score, median (P25-P75)			18.5 (16–20)		
RA patients					
Disease Duration (Years), median (P25-P75)		5.4 (2.4–17)			11.8 (1.4–33)
DAS28CRP, median (P25-P75)		2.75 (1.9–3.8)			3.4 (3–4.2)
Anti-CCP positive (%)/RF positive (%)		80/80			90/90
Erosive disease		60%			90%
No treatment (%)/DMARDs (%)/Biological treatment (%)		10/70/50			0/80/50

P25-P75, percentile 25th - percentile 75th; NA = not available; CRP, C-reactive protein; HRCT, High Resolution Computerized Tomography; FVC, Forced Ventilatory Capacity; TLC, Total Lung Capacity; FEV_1_, Forced Expiratory Volume in 1 s; DLCOc, Diffusion capacity of the Lung for Carbon monoxide corrected for hemoglobin level, GINA, Global Initiative for Asthma; ACT, Asthma Control Test Score; DAS28CRP, Disease Activity Score 28 joints combined with CRP value; CCP, cyclic citrullinated peptide; RF, rheumatoid factor; DMARD, Disease Modifying Anti Rheumatic Drugs. 1) Current or former smokers; 2) More recent than 2 years; 3) Standard Asthma lung-function test without body plethysmography; 4) Most recent lungfunction tests on RA-ILD patients were without FVC and TLC, therefore not stated. 5) One patient without DLCO_c_ value; 6) All patients in anti-IgE treatment

Age and sex were not correlated to the number of CD45^+^ CD34^+^CD11b^+^ cells or number of cultured fibrocytes in the healthy individuals (*p* = 0.9 and *p* = 0.3 respectively). When including the disease groups (total *n* = 49) there is a correlation to age for both circulating fibrocytes and number of cultured fibrocytes (*r* = 0.40 *p* = 0.006 and *r* = 0.39 *p* = 0.01 respectively).

Patients with smoking status current or former had higher levels of CD45^+^ CD34^+^CD11b^+^ cells, compared to never smokers (16 versus 19 patients, *p* = 0.01). Number of cultured fibrocytes was not different (*p* = 0.09).

Figure 3a and b illustrates the CD45^+^ CD34^+^CD11b^+^ cell and mature fibrocyte levels in patients according to disease category. ILD and RA-ILD patients had significantly higher levels of CD45^+^ CD34^+^CD11b^+^ cells compared to healthy controls (HC) (Fig. 3a, p=0.01 and *p* = 0.001 respectively). While there was no significant difference for RA or the severe asthma group compared to HC (*p* = 0.26 and *p* = 0.06 respectively). Further, there were no significant differences between the different disease categories (e.g. RA vs. RA-ILD; *p* = 0.14).

RA, severe asthma and RA-ILD all had higher levels of mature fibrocytes compared to HC (Fig. 3b, all *p* < 0.05). No significant difference was seen between HC and ILD (*p* = 0.08) or between the different disease categories.

Further, there was a significant linear correlation between measured CD45^+^ CD34^+^ CD11b^+^ cells and the number of counted mature fibrocytes per 10^5^ added monocytes in culture was *r* = 0.81, *p* < 0.001 (Fig. 3c). Correlation between CD45^+^CD34^+^CD11b^−^ and number of the cultured mature fibrocytes was insignificant (*r* = 0.17; *p* = 0.31).

Within the individual disease groups, levels of CD45^+^ CD34^+^CD11b^+^ cells and mature fibrocytes correlated significantly within HC, RA, ILD and RA-ILD but not in patients with severe asthma (HC (*r* = 0.76, *p* = 0.01), RA (*r* = 0.70 *p* = 0.02), severe asthma (*r* = 0.56, *p* = 0.15), ILD (*r* = 0.83, *p* = 0.01), RA-ILD (*r* = 0.85, *p* = 0.003).

### Association with disease activity

In the combined group of RA, severe asthma, ILD and RA-ILD patients, the level of cultured fibrocytes and CD45^+^ CD34^+^CD11b^+^ cells was significantly higher in the patients with normal C-reactive protein (CRP) (<10 mg/L) than in patients with elevated CRP (both *p* = 0.001). Median CRP in the patients with normal CRP was 2.7 mg/L (P25-P75: 1.9–4.7) and in the patients with elevated CRP 28 mg/L (P25-P75: 16–43). CD45^+^ CD34^+^CD11b^+^ cells and level of mature fibrocytes was significantly increased in the group with elevated monocytes (both *P* = 0.03). Monocyte levels was correlated to CD45^+^ CD34^+^CD11b^+^ cell levels (*r* = 0.3 *p* = 0.04), but not with number of mature fibrocytes (*r* = 0.24 *p* = 0.11).

**Fig. 4:**
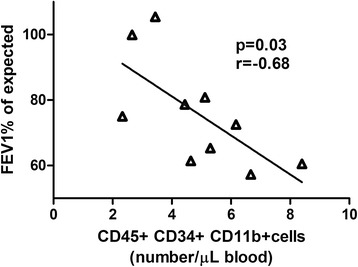
Correlation of circulating fibrocytes with forced expiratory volume in 1 s (FEV_1_), in asthma patients

In the severe asthma group the level of CD45^+^ CD34^+^CD11b^+^ cells correlated to decline in forced expiratory volume in 1 s (FEV_1_) (*r* = −0.68 *p* = 0.03) (Fig. 4). Fibrocyte levels did not correlate to Asthma Control Test Score (*p* = 0.9).

In the RA patients (combined RA and RA-ILD group), disease activity (DAS28CRP) was not correlated with level of CD45^+^ CD34^+^CD11b^+^ cells or cultured mature fibrocytes (*r* = −0.17, *p* = 0.47 and *r* = −0.31, *p* = 0.20 respectively). Further, RA patients not in remission (DAS28CRP > 2.6) did not have increased levels of CD45^+^ CD34^+^CD11b^+^ cells or number of fibrocytes in culture (*p* = 0.9 and *p* = 0.8). The same pattern appeared when comparing patients with moderate to high disease activity (DAS28CRP > 3.2) versus the rest (data not shown). Anti-cyclic citrullinated protein antibody (Anti-CCP) or IgM-Rheumatoid factor (RF) positivity did not correlate to the level of CD45^+^ CD34^+^CD11b^+^ cells or number of mature fibrocytes (all *p* > 0.2). Ninety percent of patients in the RA-ILD group were both anti-CCP and RF positive, the last solely RF positive. The RA-ILD group were not older or had longer disease course compared to the RA group (*p* = 0.15 and *p* = 0.57 respectively).

Based on a radiological pattern recognition on a high-resolution computed tomography (HRCT) of the thorax the RA-ILD patients included had the following ILD subtypes: non-specific interstitial pneumonia (NSIP) = 4, bronchiolitis obliterans (BO) = 3, hypersensitive pneumonitis (HP) = 2, usual interstitial pneumonia (UIP) = 1. In addition, four of the RA-ILD patients had their diagnosis histological confirmed on basis of surgical lung (*n* = 3) or bronchoscopic lung cryobiopsy (*n* = 1).

Prednisolone treated RA patients did not have higher levels of either CD45^+^ CD34^+^CD11b^+^ cells or number of mature fibrocytes, compared to non-prednisolone treated (*p* = 0.5 and *p* = 0.2). 85% of RA patients had erosive disease, although this group did not have higher levels of fibrocytes (*p* = 0.4 and *p* = 0.3 respectively). Methotrexate treated RA patients had a median of 4.4 CD45^+^ CD34^+^CD11b^+^ cells/μL and 3366 mature fibrocytes/10^5^ monocytes (*n* = 11) versus untreated with 6.2 CD45^+^ CD34^+^CD11b^+^ cells/μL and 6368 mature fibrocytes/10^5^ monocytes (*p* = 0.16 and *p* = 0.05 respectively).

**Fig. 5:**
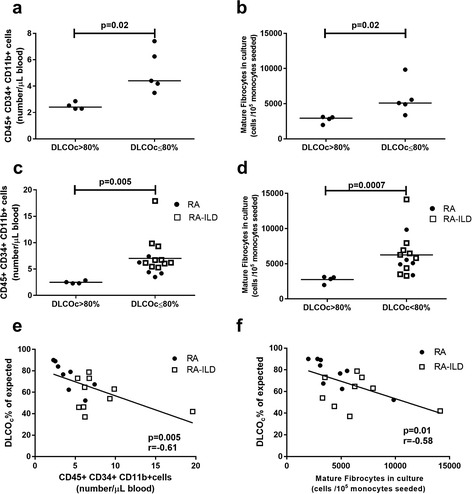
Fibrocytes and diffusion capacity. **a** Comparison of circulating fibrocytes between RA patients with high or low hemoglobin corrected diffusion capacity. **b** Comparison of cultured mature fibrocytes between RA patients with high or low hemoglobin corrected diffusion capacity. **c** Comparison of circulating fibrocytes between all RA patients, RA patients with ILD included, with high or low hemoglobin corrected diffusion capacity. **d** Comparison of cultured mature fibrocytes between all RA patients, RA patients with ILD included, with high or low hemoglobin corrected diffusion capacity. **e** Correlation of circulating fibrocytes with hemoglobin corrected diffusion capacity in all RA patients including RA-ILD patients. **f** Correlation of cultured mature fibrocytes with hemoglobin corrected diffusion capacity in all RA patients, the RA patients with ILD included

All patients in the RA-ILD and ILD groups had pathological reduced DLCO_c_ and signs of interstitial lung disease and/or fibrosis on high resolution computed tomography (HRCT). In the RA group not diagnosed with ILD, 5/9 (55%) had reduced DLCO_c_ which was associated with a 51% higher levels of circulating fibrocytes and 52% higher count of mature fibrocytes, compared to RA patients with normal DLCO_c_ (both *p* = 0.02) (Fig. 5a and b). In the combined RA and RA-ILD group the same patterns were observed (Fig. 5c *p*=0.005 and *p* = 0.0007 respectively). Among all RA patients the level of circulating and mature fibrocytes correlated to decline in DLCO_c_. (*r*=−0.61 *p* = 0.005 and *r* = −0.58, *p* = 0.01 respectively) (Fig. 5e and f).

Only one RA patient in the group not diagnosed with ILD, had a reticular pattern on chest X-ray as an indicative of potential fibrosis but without HRCT clarification. The findings on chest X-ray versus without, did not correlate with increased CD45^+^ CD34^+^CD11b^+^ level or number of cultured fibrocytes (*p* = 0.3 and *p* = 0.11 respectively). In the RA-ILD group all had radiological signs of ILD on HRCT and 6/10 on chest x-ray. There was no difference in fibrocyte levels in RA-ILD patients with or without signs of ILD on chest X-ray (*p* = 0.2 and *p* = 0.8 respectively).

## Discussion

We have proven that peripheral blood CD45^+^CD34^+^CD11b^+^ (7-AAD^−^CD3^−^ CD19^−^CD294^−^) cells are circulating fibrocytes, per definition as they express pro-collagen type 1. For the first time, we show that the level of circulating fibrocytes in peripheral blood correlates with the number of monocytes that in vitro differentiate into mature fibrocytes. Further, levels of circulating fibrocytes were elevated in patients with ILD and RA-ILD compared to HC. In patients with severe asthma, the level of circulating fibrocytes correlated with reduction in FEV1. As far as we know, we report, for the first time, that higher levels of both mature and circulating fibrocytes in RA and RA-ILD patients are associated with reduced DLCO_c_.

Previous studies have shown high levels of circulating fibrocytes in IPF that correlated to disease severity and in one study mortality [24, 25]. In accordance, we found increased levels of circulating fibrocytes in the ILD group, consisting of IPF and fibrotic NSIP patients, compared to HC. All ILD patients had signs of lung fibrosis on their chest X-rays compatible with their severely reduced DLCO_c_ (median 36% of predicted). To better understand the role of fibrocytes in the ILD group, studies of larger cohorts are required, including patients at different disease stages. In this regard, the results from the ongoing PROFILE multicenter prospective study on ILD (NSIP and IPF) (Clinical trials NCT01134822), in which the fibrocyte levels are studied prospectively according to pulmonary function test and radiographic data, are awaited.

Increased numbers of circulating fibrocytes have been observed in murine RA models [4]. We found higher levels of circulating fibrocytes in the RA-ILD group compared to HC, not in the RA group not previously diagnosed with ILD. We found increased levels of in vitro matured fibrocytes in both RA and RA-ILD patients compared to HC. The levels of mature or circulating fibrocytes were not increased in patients with elevated RA disease activity. Thus we confirm the findings from a recent study reporting that an elevated levels of circulating fibrocytes (measured by traditional intracellular collagen staining) in RA patients is unassociated to disease activity [26]. An earlier publication from the same study group found that the extent of fibrocyte activation in six RA patients, as measured by phosphorylation of defined signaling effectors, did correlate with RA disease activity [27].

The present study shows that the level of monocytes differentiating to mature fibrocytes is elevated in RA and RA-ILD, compared to HCs. Whereas fibrocyte levels has previously been found elevated in synovial fluid in RA, the level in RA-ILD patients has not been investigated previously to our knowledge [26]. In the RA group, the levels of circulating and mature fibrocytes were increased in the group with pathological reduced DLCO_c_ (See Fig. 5a–d). To further investigate this relationship in RA, prospective cohort studies following fibrocyte levels and pulmonary function test combined with HRCT data are warranted.

Overall, the levels of circulating or number of mature fibrocytes were not increased in the subgroup of patients with elevated CRP. In a previous in vitro study adding increasing the doses of CRP did not inhibit fibrocyte differentiation [28]. Elevated leukocyte levels were accompanied with increased levels of both circulating and mature fibrocytes. This suggests that circulating fibrocytes cannot be used as a traditional inflammation marker.

The traditional method for measuring circulating fibrocytes is to first isolate PBMCs and hereafter perform permeabilization and fixation for intracellular staining [22]. This method involves several washing steps, is time consuming, costly and requires large volumes of venous blood, which makes it difficult to use in daily clinical practice (see Table 3 for comparison of the two methods). Further, a measurement of the absolute fibrocyte concentration in peripheral blood is not possible when using isolated PBMCs. Therefore, the method introduced by Bianchetti et al.*,* where only surface markers were used and 100 μL of venous blood was sufficient for quantitative fibrocyte measurements, was a marked improvement. For a comparison between the traditional method of measuring circulating fibrocytes and the method using only surface markers please see Table 3.

**Table 3:** Comparison of methods to measure circulating fibrocytes in peripheral blood

	Traditional	New method
Material	Peripheral blood	Peripheral blood
Volume needed	10 mL	100 μL
Processing	Isolate PBMC by density centrifugation (Ficoll-Paque) ⇩ Extracellular antibodies added ⇩ Cell fixation and permabilization ⇩ Intracellular antibodies added	Add antibodies and incubate. ⇩ Red blood cell lysis solution added
Cell washing steps	8	0
Detection method	Flow cytometry	Flow cytometry
Fibrocyte Concentration	Estimate percentage of total PBMC or per mL of blood	Absolute concentration using beads
Processing time	Over 3 h	Under 1 h
Advantages	Unused isolated PBMC can be used for other purposes (e.g. culture or stored)	Fast and small blood volume Reduced cell loss due to no washing steps or permabilization
Reference	[22]	Method section or [13]

In this study, we have simplified this method further by omitting the antibodies against CD16, CD115 and CD20 in the flow cytometric panel [13]. CD16 positive cells should not in general be discarded in the flow cytometry analysis, as mature fibrocytes can be CD16 positive, and cultures of CD14^+^CD16^+^ monocytes and CD14^+^CD16^−^ monocytes have been shown to differentiate to the same level of mature fibrocytes [29, 30]. Further, isolated CD34^+^CD16^+^ cells cultured with IL-4 established spindle shaped morphology and collagen expression, thereby fulfilling the criteria for being fibrocytes [23]. In IPF, levels of peripheral blood monocytes with high expression of CD14^+^ CD16^+^ at baseline was associated with a worse outcome in a prospective study, also suggesting a role of CD16^+^ cells in the fibrotic disease [20]. A potential bias could here be contamination by CD16^+^ neutrophils, which was solved by the gate in the initial FSC-SSC plot (See Fig. 1). CD115 and CD20 was omitted as negative selection markers in our flow cytometry analysis, as we found only 3 and 1% of the CD45^+^ CD34^+^ CD11b^+^ cells, respectively, to be positive for these markers. The presented flow cytometric method is quick and stable with a low CV% of 9%, based on the very low absolute fibrocyte count in healthy subjects (median of 1.4 fibrocytes/μL) and low data spread (P25-P75 of 1.2–1.5 fibrocytes/μL). Further, the blood sample can be stored for 24 h at room temperature without significantly affecting the fibrocyte measurement, making the method easy to adapt in daily clinical work.

Several findings in this study support that the fast and stable flow cytometric method measures circulating fibrocytes. First, after stimulation with IL-4 for 2 days we found that the majority of CD45^+^ CD34^+^ CD11b^+^ cells were pro-collagen type 1 positive (90.2% (SD ±1.5%)), compared to only 6.3% (SD ±4.6%) of the corresponding CD11b^−^ cells. In agreement, a similar finding by Bianchetti et al. showed that over 88% of sorted cells from 6 asthma patients had collagen 1 expression, using stimulation with endothelin 1 (ET-1) [13]. For further evidence, we tested the CD45^+^CD34^+^CD11b^+^ population for CXCR4 expression, which is used as a fibrocyte marker in several studies [2, 14], and found a mean frequency of 75% (SD ± 9%) of the cells expressed CXCR4. Further, in our study the isolated procollagen type 1 positive CD45^+^CD34^+^CD11b^+^ cells resembled monocytes in appearance and size, given further support as fibrocytes are derived from a monocyte subset (See Fig. 2c). To further validate, the flow cytometry results of the panel was compared with a distinct and well validated method for estimating level of circulating cells differentiating to fibrocytes [16, 31, 32]. This resulted in a significant correlation (*r* = 0.81), between the measured level in peripheral blood of CD45^+^CD34^+^CD11b^+^ cells and the number of mature fibrocytes (Fig. 3c). This further strengthens the validity of the presented flow cytometric method.

Further, we included patients with severe asthma corresponding to GINA level 5, and found that the level of circulating fibrocytes correlated to FEV_1_ decline (*r* = −0.68 *p* = 0.03) (Fig. 4). This is in agreement with previous findings of correlation between fibrocytes, defined predominantly by intracellular collagen staining, and asthmatic activity, further supporting the validity of the presented flowcytometric panel [13–15]. In the case of severe asthma, a potential explanation of the increased fibrocyte levels could be due to an ongoing remodeling process in the small airways which also leads to reduced ventilation values on pulmonary function tests.

Several factors have been found to influence the in vitro transformation of monocytes to mature fibrocytes including CD4^+^ T-cell interaction, endothelin, serum amyloid protein (SAP), TNF-α, thrombin, IL-4, IL-17 and IL-22. [6, 7]. IL-4 level is elevated in ILD and asthma lung biopsies and in peripheral blood and synovial fluid in early RA [8, 15, 19, 26]. The method used for in vitro culturing fibrocytes, where IL-4 is added, could therefore potentially be a method to estimate number of cells with potential to differentiate to mature fibrocytes in the diseased organs. When culturing mature fibrocytes, adding IL-4 to the serum free media at the concentration (5 ng/ml) used in the present study, has previously been shown to result in a stimulation of fibrocyte differentiation of around 200% compared to serum free media without IL-4 [33].

A study limitation is that the sorted cells were not stained without culture, or cultured for 5 days to investigate whether the majority transformed to mature fibrocytes. Culturing was stopped after 2 days due to signs of cell distress after the cell sorting procedure. Further, the study population is small (*n* = 49), and although we did find significant clinical correlations, this needs exploration in larger cohorts. The culturing of mature fibrocytes was most difficult in the ILD group, with frequent signs of cell distress on the stained cells. The majority of ILD patients was elderly and had end stage lung disease, which could have affected the in vitro cell viability. The number of cells with potential to differentiate into mature fibrocytes could therefore be underestimated in this group. Further, data on DLCO_c_ in the asthma patients was lacking, although this parameter is usually normal or elevated in asthma [34]. Additionally, 6/49 patients did not have recent radiographic data <2 year, and none of the RA patients (not diagnosed with ILD) had undergone a HRCT. As HRCT is the first choice radiographic modality when suspecting ILD, a potential drawback could be that the study has underestimated radiological fibrosis patterns in the RA group not diagnosed with ILD [1]. In addition, there was a profound variation in length of disease (1.4–33 years) and treatment strategy in the RA category.

In general, it is estimated that 20% of RA patients develop ILD. In the present study 56% of RA patients and 100% of the RA-ILD patients had reduced DLCO_c_. The RA-ILD group, was composed of several different ILD subtypes (NSIP 40%, UIP 10%, BO 30% and HP 20%). A study with the power to analyze fibrocyte levels and activation in the different RA-ILD subgroups, could potentially provide a deeper understanding of the role of fibrocytes and the difference in pathogenesis of these RA-ILD subtypes.

This result is consistent with a recent and major retrospective study on 550 RA patients, which found that 43% of these patients had or were about to develop RA-ILD [35]. In the study, the RA-ILD diagnosis was based on HRCT, and patients with RA-ILD had a significantly and expected DLCO reduction compared to other RA patients. Not all RA patients develop RA-ILD, and to identify factors protecting against development of lung fibrosis is crucial. Especially the potential role of smoking, linked to fibrocyte levels in the present study, and anti-CCP positivity, which we did not find linked to fibrocyte level, deserves further investigation. The role of Methotrexate in treatment of RA-ILD is currently unclear and also deserves further study [1]. We found a trend toward lower levels of both circulating and number of mature fibrocytes in Methotrexate treated versus untreated RA patients, although not significant (*p* = 0.16 and *p* = 0.05 respectively).

We are currently conducting a larger prospective RA study with newly diagnosed and untreated RA and longstanding RA patients to further study the role of fibrocytes in RA-ILD.

## Conclusions

We presented a fast and valid method for measuring circulating fibrocytes using flow cytometry on lysed peripheral blood. Further, we showed for the first time, that the level of circulating fibrocytes correlated with the number of mononuclear cells that differentiated into mature fibrocytes in vitro. Reduced DLCO_c_ was correlated with high levels of circulating and mature fibrocytes in RA, which has not been reported previously. In such, this study suggests that fibrocytes may exhibit an important role in the pathogenesis of RA-ILD, which requires further clarification in future studies.

## Additional file

Additional file 1: Title of data: Detailed information regarding the presented methods and data on the additional antibodys tested. Description of data: **Table S1.** contains detailed information on the flow cytometry, cell culture and cell sorting procedures used. **Table S2.** contains data on the surface expression of the addtional antibodys tested. (DOCX 21 kb)

## Abbreviations

ACT: Asthma control test score
Anti-CCP: Cyclic citrullinated protein antibody
BO: Bronchiolitis obliterans
CRP: C-reactive protein
DAS28CRP: Disease Activity Score 28 joints combined with CRP
DLCO_c_: Diffusion capacity of the lung for carbon monoxide adjusted for hemoglobin level
DMARD: Disease modifying anti rheumatic drugs
DPLD: Diffuse parenchymal lung diseases
EDTA: Ethylenediaminetetraacetic acid
ET-1: Endothelin 1
FACS: Fluorescence activated cell sorting
FEV_1_: Forced expiratory volume in 1 second
FMO: Fluoroscence minus one
FVC: Forced ventilatory capacity
GINA: Global initiative for asthma
HP: Hypersensitivity pneumonitis
HRCT: High resolution computed tomography
IL4: Interleukin 4
ILD: Interstitial lung disease
IPF: Idiopathic pulmonary fibrosis
NEAA: Non-essential amino acids
NSIP: Non-specific interstitial pneumoniatis
PBMC: Peripheral blood mononuclear cells
RA: Rheumatoid arthritis
RF: Rheumatoid factor
TLC: Total lung capacity
UIP: Usual interstitial pneumonia
