# Supplementary material for: Fibrocyte measurement in peripheral blood correlates with number of cultured mature fibrocytes in vitro and is a potential biomarker for interstitial lung disease in Rheumatoid Arthritis
Source: Respir Res. 2017 Jul 18;18:141. doi: 10.1186/s12931-017-0623-9 (PMC5516315; doi:10.1186/s12931-017-0623-9)
Supplement: Additional file 1: — Title of data: Detailed information regarding the presented methods and data on the additional antibodys tested. Description of data: Table S1. contains detailed information on the flow cytometry, cell culture and cell sorting procedures used. Table S2. contains data on the surface expression of the addtional antibodys tested. (DOCX 21 kb) [file 12931_2017_623_MOESM1_ESM.docx]

**Table S1**

| **Before flow** | | | | | | |
| --- | --- | --- | --- | --- | --- | --- |
|  | **Product** | **Dose/test** | **Total volume** |  | **Manufacturer** | **Product** |
|  | High-Yield Lyse | 2 ml | 500 ml |  | Thermo Fisher, Waltham, USA | HYL250 |
|  | Lymphoprep |  |  |  | StemCell, Grenoble, France | 7851 |
|  | TrueCount tubes |  |  |  | BD Bioscience, San Jose, USA | 340334 |
| **Flowcytometry** | | | | | | |
|  | **Target** | **Dose/ µL** | **Flurocrome** | **Clone** |  |  |
|  | CD45 | 5 | V500-C | 2D1 | BD Bioscience, San Jose, USA | 655873 |
|  | CD34 | 5 | PE | 581 | BD Bioscience, San Jose, USA | 555822 |
|  | CD11b | 5 | Pacific Blue | ICRF44 | BD Bioscience, San Jose, USA | 558123 |
|  | CD19 | 2.5 | PerCP-Cy5.5 | HIB19 | BD Bioscience, San Jose, USA | 561295 |
|  | CD3 | 2.5 | PE-Cy5 | HIT3a | BD Bioscience, San Jose, USA | 555341 |
|  | CD294 | 2.5 | PerCP-Cy5.5 | BM16 | BD Bioscience, San Jose, USA | 561660 |
|  | Via-Probe (7-AAD) | 2 | 7-AAD |  | BD Bioscience, San Jose, USA | 555815 |
|  | **Other tested surface markers** | |  |  |  |  |
|  | CD16 | 7.5 | FITC | B73.1 | BD Bioscience, San Jose, USA | 561308 |
|  | CD184 (CXCR4) | 5 | APC | 12G5 | BD Bioscience, San Jose, USA | 555976 |
|  | CD115 | 5 | Alexa Fluor 647 | 94D21E4 | BD Bioscience, San Jose, USA | 564945 |
|  | CD20 | 1.25 | PerCP-Cy5.5 | 2H7 | BD Bioscience, San Jose, USA | 560736 |
|  | **Isotype** |  |  |  |  |  |
|  | CD45 | 5 | V500-C |  | BD Bioscience, San Jose, USA | 560787 |
|  | CD34 | 5 | PE |  | BD Bioscience, San Jose, USA | 555749 |
|  | CD11b | 5 | Pacific Blue |  | BD Bioscience, San Jose, USA | 558120 |
|  | CD184 (CXCR4) | 5 | APC |  | BD Bioscience, San Jose, USA | 555576 |
| **Validation** | | | | | | |
|  | Pro-Collagen type 1 | 1/150 |  | 2Q576 | Abcam, Cambridge, UK | ab64409 |
|  | RoboSep CD34 Kit |  |  |  | StemCell, Grenoble, France | 15086RF |
|  | **CellMedia after isolation 100 ml** | |  |  |  |  |
|  | Hanks media |  | 90 ml |  | Sigma-Aldrich, St. Louis, USA | H6648 |
|  | HSA | 50 mg/ml | 5 mg/ml (10 ml) | | Sigma-Aldrich, St. Louis, USA |  |
|  | EDTA | 0.5 M | 2 mM (0,4 ml) | | Sigma-Aldrich, St. Louis, USA |  |
|  | **FACS** | | | | | |
|  | CD34 | 20 | PE | 8G12 | BD, New Jersey, USA | 340667 |
|  | **Fibrocyte culture media 50 ml** | | | | | |
|  | **Component** | **Added/ ml** |  |  |  | **Product** |
|  | FibroLife Medium | 47 |  |  | LifeLine Cell Technology, Frederick, USA | LL-0001 |
|  | IL-4 | 2.5 µL (at 100 µg/ml) | |  | CellSystems, Troisdorf, Germany | CS-C1064 |
|  | 10 mM HEPES | 0.5 |  |  | Sigma-Aldrich, St. Louis, USA |  |
|  | Non-essential amino acids (NEAA) | 0.5 |  |  | Sigma-Aldrich, St. Louis, USA |  |
|  | Sodium pyruvate (1 mM) | 0.5 |  |  | Sigma-Aldrich, St. Louis, USA |  |
|  | 2 mM glutamine | 0.5 |  |  | Sigma-Aldrich, St. Louis, USA |  |
|  | Pen-Strep (100 U/ml penicillin/100 ug/ml streptomycin) | 0.5 |  |  | Sigma-Aldrich, St. Louis, USA |  |
|  | ITFS-3 | 0.5 |  |  | Sigma-Aldrich, St. Louis, USA |  |

**Table S2**

| **Antibody**  **tested** | n | Fraction of CD45^+^ CD34^+^ CD11b^+^ cells expressing surface marker  (mean ± SD) | Fraction of CD45^+^ CD34^+^ CD11b^-^cells  expressing surface marker  (mean ± SD) | *p*-value |
| --- | --- | --- | --- | --- |
| CXCR4 | 13 | 75 ± 9 | 52 ± 13 | 0.009 |
| CD16 | 13 | 45 ± 12 | 1.4 ± 1.7 | <0.001 |
| CD115 | 13 | 3 ± 5 | 4 ± 5 | NS |
| CD20 | 19 | 0.9 ± 1.8 | 3.5 ± 3.7 | NS |

NS=not significant.
